# Challenges of synthesizing medical education research

**DOI:** 10.1186/s12916-014-0193-3

**Published:** 2014-10-29

**Authors:** Rachel H Ellaway

**Affiliations:** Northern Ontario School of Medicine, 935 Ramsey Lake Road, Sudbury, Ontario P3E 2C6 Canada

**Keywords:** Systematic review, Medical education, Evidence-based practice, STORIES framework

## Abstract

The expectation that the primary function of systematic reviews in medical education is to guide the development of professional practice requires basic standards to make the reports of these reviews more useful to evidence-based practice and to allow for further meta-syntheses. However, medical education research is a field rather than a discipline, one that brings together multiple methodological and philosophical approaches and one that struggles to establish coherence because of this plurality. Gordon and Gibbs have entered the fray with their common framework for reporting systematic reviews in medical education independent of their theoretical or methodological focus, which raises questions regarding the specificity of medical education research and how their framework differs from other systematic review reporting frameworks. The STORIES (STructured apprOach to the Reporting In healthcare education of Evidence Synthesis) framework will need to be tested in practice and potentially it will need to be adjusted to accommodate emerging issues and concerns. Nevertheless, as systematic reviews fulfill a greater role in evidence-based practice then STORIES or its successors should provide an essential infrastructure through which medical education syntheses can be translated into medical education practice.

Please see related article: http://www.biomedcentral.com/1741-7015/12/143.

## Background

As a field of research matures, the question arises: what to do with the accumulating published knowledge and evidence it has generated? This is particularly important for fields of research that explore the professions and their practices, such as research into medical education where research typically has a direct relationship with quality improvement. The expectation that we move from opinion- to evidence-based practice is compelling and widespread [1,2] and this has been reflected in medical education by the growing number of systematic reviews and by movements including, but not limited to, the Best Evidence Medical Education (BEME) initiative that has overseen the development of a number of systematic reviews as well as the development and dissemination of systematic review practices [3].

However, it is arguable, at least in medical education, that there has been a lot more said about how systematic reviews should be conducted than there has been on the role of reviews in the medical education literature or on their relationship to practice [4]. This is a particular issue in medical education because the systematic review can function both as an evidence-based guide to a particular topic and as a summative statement of how the topic has been considered and explored up to the time of the review. However, if the review were simply to be an intellectual artifact then there would be little need for reporting frameworks as much of a review’s value would come from being situated in a particular time, paradigm, and rhetoric. The expectation that the primary function of a systematic review is to guide the development of professional practice requires a greater attention to what a review, or at least the outputs of a review, should include. Indeed, it is one of the key features of systematic reviews that they follow defined protocols and systematic processes of selecting and reviewing papers [3] as well as reporting their findings in support of evidence-based practice.

### Herding cats

Systematic review reporting frameworks have been developed to establish basic standards to make reports of reviews more useful to evidence-based practice and to allow for further meta-syntheses. These frameworks have tended to be methodology-specific, such as PRISMA (Preferred Reporting Items for Systematic Reviews and Meta-Analyses) [5] and RAMESES (Realist and MEta-narrative Evidence Syntheses: Evolving Standards) [6], reflecting a broad consensus that the primary unifying construct for reviews should be the methods they use. However, as Dornan *et al*. (referencing Kelly and Murray) note: ‘education researchers change practice within a system that is open, complex, non-linear, organic, and historical and use qualitative as well as quantitative methods to evaluate the outcome’ [7]. Medical education research should, therefore, be seen as a field rather than a discipline, one that brings together multiple methodological and philosophical approaches and, in doing so, it struggles to establish coherence because of this intrinsic plurality [8]. As a result, systematic reviews in medical education tend not to share a common methodological stance: some reviews are meta-analyses of experimental results seeking an optimal form of practice while others may take the form of explanatory narrative realist reviews of what works [9] and how it works in different contexts [10]. This can be challenging if one expects uniformity in systematic reviews and it can be particularly confusing to clinical teachers who do not have a strong basis in the academic discourses of medical education scholarship. It can also be challenging in reporting a review to balance its anticipated utility with the disciplinary style it follows. Add to this the vanishing returns on systematicity in reviews [11] then what may at first have seemed like a simple task, to systematically review evidence in medical education, becomes far more complicated. This is where Gordon and Gibbs have entered the fray with their common framework for reporting systematic reviews in medical education independent of their theoretical or methodological focus [12].

### The STORIES framework

This is a bold step and in taking it the authors raise questions regarding the specificity of medical education research and how their STORIES (STructured apprOach to the Reporting In healthcare education of Evidence Synthesis) framework differs from other systematic review reporting frameworks, particularly as there are many elements within STORIES that are common to other frameworks. Their solution challenges assumptions that the systematic review is essentially a generic technique (or repertoire of techniques) that can be applied to any topic or body of evidence. The STORIES framework requires reviewers to look instead at the methodologies employed in the studies reviewed, the methodology of the review itself, and the intended uses of the review.

Evidence-based practice in medicine has been identified as having a number of shortcomings, including a failure to address reporting bias and vested interest, particularly in privileging statistical and algorithmic approaches [13]. If a review is to be used for evidence-based practice then it should involve a degree of reflexivity regarding the nature of evidence-based practice in medical education and how it can be best supported and advanced, not least because in medical education, with its plural and discontinuous methodologies and philosophies, this can sometimes be more about the pursuit of ‘least-worst evidence’ or ‘best available evidence’ rather than what might truly be considered ‘best evidence’. There should, therefore, also be a consideration of the relationship between the nature of evidence and the phenomena under consideration [14].

## Conclusions

The STORIES framework will need to be tested in practice and it will most likely need to be adjusted to accommodate emerging issues and concerns. The development and adoption of STORIES provides an opportunity to reflect on the role of systematic reviews in medical education and their place in the rhetoric and philosophy of evidence. In summary, as medical education research matures as a field and as systematic reviews become more common and fulfill a greater role in evidence-based practice, STORIES or its successors have the potential to provide an essential infrastructure through which medical education research can be translated into medical education practice.

## Abbreviations

BEME: Best Evidence Medical Education
PRISMA: Preferred Reporting Items for Systematic Reviews and Meta-Analyses
RAMESES: Realist and MEta-narrative Evidence Syntheses: Evolving Standards
STORIES: STructured apprOach to the Reporting In healthcare education of Evidence Synthesis

## References

[1] Davies P (2000). Approaches to evidence-based teaching. Med Teach.

[2] Van Der Vleuten CP, Dolmans DH, Scherpbier A (2000). The need for evidence in education. Med Teach.

[3] Albanese M, Norcini J (2002). Systematic reviews: what are they and why should we care?. Adv Health Sci Educ.

[4] Cook DA, West CP (2012). Conducting systematic reviews in medical education: a stepwise approach. Med Educ.

[5] Moher D, Liberati A, Tetzlaff J, Altman DG, The PRISMA Group (2009). Preferred reporting items for systematic reviews and meta-analyses: The PRISMA Statement. BMJ.

[6] Wong G, Greenhalgh T, Westhorp G, Buckingham J, Pawson R (2013). RAMESES publication standards: realist syntheses. BMC Med.

[7] Dornan T, Littlewood S, Margolis SA, Ypinazar V, Scherpbier A, Spencer J (2007). Identification of best evidence in medical education. Case study. Med Teach.

[8] Regehr G (2004). Trends in medical education research. Acad Med.

[9] Cook DA, Hatala R, Brydges R, Zendejas B, Szostek JH, Wang AT, Erwin P, Hamstra S (2011). Technology-enhanced simulation for health professions education: a systematic review and meta-analysis. JAMA.

[10] Thistlethwaite JE, Bartle E, Chong AA, Dick ML, King D, Mahoney S, Papinczak T, Tucker G (2013). A review of longitudinal community and hospital placements in medical education: BEME Guide No. 26. Med Teach.

[11] Eva KW (2008). On the limits of systematicity. Med Educ.

[12] Gordon M, Gibbs T (2014). STORIES statement: publication standards for healthcare education evidence synthesis. BMC Med.

[13] Greenhalgh T, Howick J, Maskrey N (2014). Evidence based medicine: a movement in crisis?. BMJ.

[14] Thistlethwaite J, Davies H, Dornan T, Greenhalgh T, Hammick M, Scalese R (2012). What is evidence? Reflections on the AMEE symposium, Vienna, August 2011. Med Teach.

